# Visible Light Curable Restorative Composites for Dental Applications Based on Epoxy Monomer

**DOI:** 10.3390/ma7010554

**Published:** 2014-01-20

**Authors:** Alessandra Vitale, Marco Sangermano, Roberta Bongiovanni, Peter Burtscher, Norbert Moszner

**Affiliations:** 1 Politecnico di Torino, Department of Applied Science and Technology, C.so Duca degli Abruzzi 24, Torino 10129, Italy; E-Mails: marco.sangermano@polito.it (M.S.); roberta.bongiovanni@polito.it (R.B.); 2 Ivoclar Vivadent AG, Macromolecular Chemistry & Synthesis, Bendererstrasse 2, Schaan FL-9494, Liechtenstein; E-Mails: peter.burtscher@ivoclarvivadent.com (P.B.); norbert.moszner@ivoclarvivadent.com (N.M.)

**Keywords:** cationic photopolymerization, visible light curing, epoxy monomer

## Abstract

A cationic photo-curable cycloaliphatic epoxy resin has been investigated as reactive monomer in blue light crosslinking process. We have demonstrated that camphorquinone is able to abstract labile hydrogen from the epoxy monomer, giving rise to the formation of carbon-centered radicals that are oxidized by the onium salt; a complete epoxy group conversion was reached after 50 s of irradiation. The presence of water up to 1 wt% was tolerated without any important detrimental effect on the kinetics of light-curing. The presence of the inorganic filler up to 65 wt% did not significantly influence the curing process.

## 1. Introduction

Many of the currently used commercially available restorative composites for dental applications are composed of a mixture of about 40–90 wt% of different surface-modified fillers and 10–60 wt% of an organic matrix [1]. Usually the organic matrix consists of a mixture of crosslinking dimethacrylates, which show a strong polymerization shrinkage ranging from 6 up to 14 vol% [2]. Shrinkage accompanies the curing as the reaction transforming a liquid monomer into a crosslinked polymer usually in the glassy state means the replacement of secondary bonds by covalent bonds, with a strong reduction of interatomic distances. The polymerization shrinkage can be reduced to a value of 2–4 vol% by the addition of fillers; however it can still cause the formation of a marginal gap during polymerization of the composite in the oral cavity [3].

In order to overcome this limitation, a strategy to reduce shrinkage is the delaying of gelation of the acrylic systems in order to reduce strain during curing [4]. This method was successfully shown and fully explained by Leterrier [5] for acrylic UV-cured formulations. An alternative strategy is the use of cyclic monomers. In fact, cyclic monomers show lower volume shrinkage because they cure by ring-opening polymerization: during this kind of polymerization for every bond that goes from a van der Waals distance to a covalent distance another bond goes from a covalent distance to a near van der Waals distance, thus counterbalancing the shrinkage accompanying the chain propagation. A number of cyclic monomers were investigated and the results are reported in different reviews [1,3,5,6].

Among cyclic monomers, cycloaliphatic epoxy monomers show a very little volume shrinkage upon irradiation [7]; therefore for this reason the application of cationic photopolymerizable epoxy-based compositions for dental fillings have found increasing attention in patent applications [8,9].

In particular, cycloaliphatic epoxy monomers are of great interest since they demonstrate significantly lower shrinkage than methacrylic resins, still showing high reactivity to be cured by cationic photopolymerization in an acceptable time frame and to an adequate depth using a visible light source.

A typical formulation contains camphorquinone (CQ) as visible light sensitizer, which is actually the most frequently used photoinitiator in dental materials cured by visible light, a diphenyl iodonium salt as photoinitiator and a low basic amine as electron-donor co-initiator [10]. For cationic photopolymerization the amine must have a low basicity in order to prevent polymerization inhibition, and the iodonium salt must have a non-nucleophilic counterion, such as hexafluoroantimonate or hexafluorophosphates [11,12].

While it has been demonstrated that visible light cationic photopolymerization of epoxides can be initiated with success using a three component initiator system, it is important at this stage to investigate the effect of the presence of a high ceramic filler content on crosslinking reaction.

In this paper, we are focusing on the investigation of the conditions to induce a blue light-curing of epoxy-based formulations for dental applications. For this reason, a silicone cycloaliphatic diepoxy resin was used as matrix. The suitable photoinitiator system composition was defined and the effect of water content and filler content was taken into account.

## 2. Results and Discussion

### 2.1. Cationic Photopolymerizable Systems

Photoinitiated ring opening polymerization of epoxy resins has been the subject of extensive studies during the last two decades [13,14]. UV-curing of such resins has been achieved since the discovery of photochemically reactive cationic initiators as iodonium salts.

**Scheme 1.**
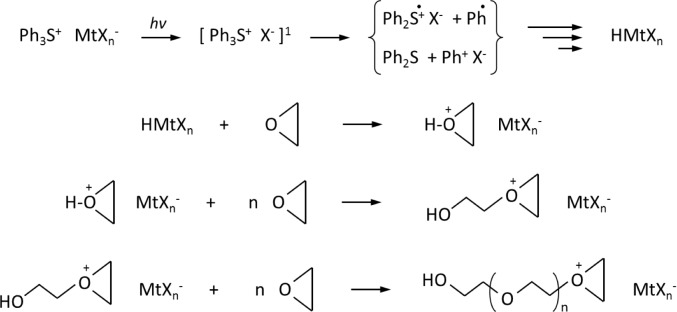
Schematic process of cationic photopolymerization of an epoxy monomer in the presence of a generic triphenyl sulfonium salt.

The initiation of cationic UV-curing polymerization is a multistep process involving first the photoexcitation of diaryliodonium or triarylsulfonium salts, and then the decay of the resulting excited singlet state with both heterolytic and homolytic cleavages. Cations and aryl-cations generated are very reactive with monomers to give the formation of a Bronsted acid, which is the actual initiator of cationic polymerization. The ring opening polymerization takes place by protonation of the monomer, followed by the addition of further monomer molecules, thus resulting in a chain growth reaction (see Scheme 1) [15].

The main problem for cationic process is related to the fact that the major absorption bands of these onium salts initiators fall in the deep UV region and do not overlap with the emission band of visible light. The strategy usually adopted consists in shifting the photoinitiator light absorption towards longer wavelengths using a photosensitizer in the presence of a iodonium salt, and obtaining the promotion of photoinduced cationic polymerization by photochemical sources of free radicals.

**Scheme 2.**
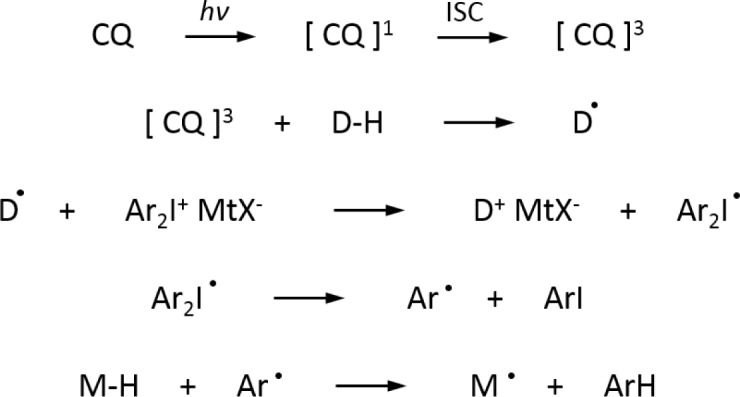
Mechanism of radical induced cationic polymerization.

In our investigated systems, we selected CQ as photosensitizer. Usually CQ is employed together with a hydrogen donor as co-iniatitor (D–H molecule in the Scheme 2): the oxidation of the formed free radicals by diaryliodonium salts produces cationic species (see Scheme 2), which subsequently initiate cationic polymerization.

CQ is well known to undergo n-π* excitation on irradiation to initially generate the excited singlet state that rapidly undergoes efficient intersystem crossing to the excited triplet. Due to the diradical character of the excited triplet carbonyl, there is a strong tendency of these species to abstract hydrogen atoms from an appropriate donor (D–H). At this point, the onium salt can oxidize this radical. The carbon-centered monomer radical M• can interact with the onium salt to induce its reduction. The generation of an aryl radical by the decomposition of the diaryliodide free radical closes the cycle by providing a species that again can abstract a hydrogen atom from the monomer.

Amines are often used as hydrogen donors. Unfortunately the basicity of the amines is a major issue when they are used as co-initiator in a cationic photopolymerization system. In fact, owing to the living character of cationic polymerization it is common to use aliphatic amines to terminate cationic chains. In this study, we wanted to polymerize under visible light an epoxy monomer; therefore besides the onium salt and the photosensitizer CQ, we needed a hydrogen donor like an amine. An aromatic amine was selected as it is characterized by lower basicity, therefore can be used, in moderate amount, even for cationic UV-curable systems.

The selected epoxy resin (UV30) shows a low cytotoxicity and no mutagenity (negative Ames test) in contrast to the other commercially available dicycloaliphatic epoxy resins. This can be explained based on the hydrophobic properties of the resin [16].

**Figure 1.**
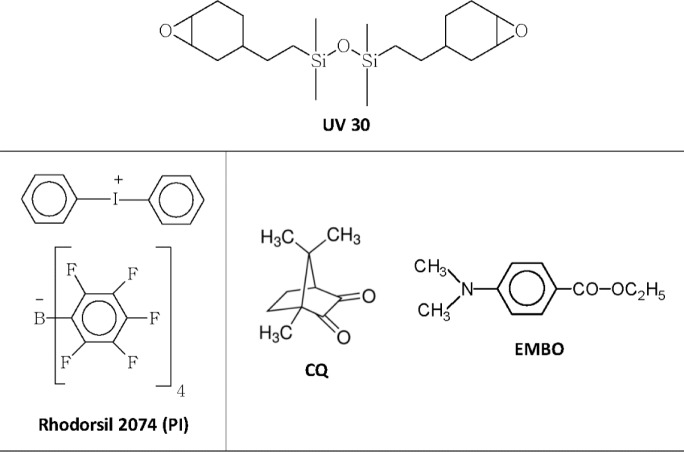
Chemical structures of the monomer and photoinitiators.

The chemical structures of the monomer and the photoinitiators [iodonium salt photoinitiator Rhodorsil 2074, photosensitizer CQ, aromatic amine ethyl 4-(dimethylamino)benzoate EMBO] used in this work are reported in Figure 1. The relative monomer and initiator concentrations for the studied systems are listed in Table 1.

**Figure 2.**
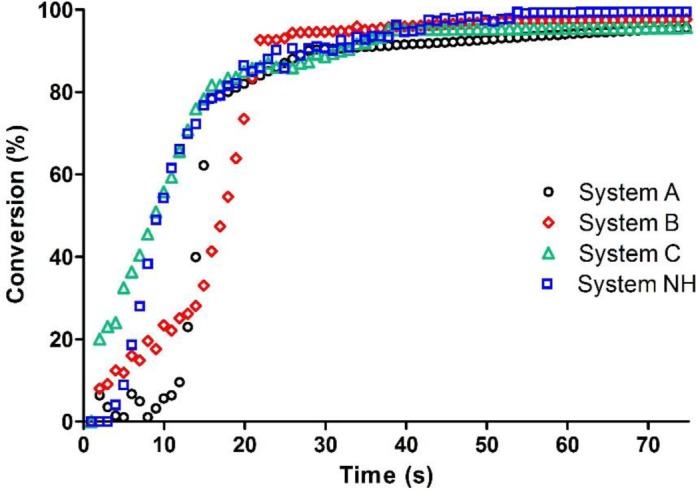
RT FT-IR conversion curves as a function of irradiation time for the epoxy based formulation containing different photoinitiator systems.

The epoxy resin in the presence of a three component initiating system made of the onium salt, the photosensitizer and the amine (System NH, Table 1) showed a reasonable initial reaction rate and a complete conversion after 40 s of irradiation with blue light (see Figure 2).

Then we examined the photopolymerization efficiency of a two component photoinitiator system eliminating the amine co-initiator from the formulations. At first we kept the same ratio between the onium salt and camphorquinone, *i.e.*, 9:1 (System A, Table 1). From the kinetics curves reported in Figure 2 it is evident that the absence of the EMBO does not affect the final epoxy group conversion, but has an important effect on reaction rate: an induction time is evident and the conversion starts with a delay of 10 s. This induction time is probably due to a lower radical rate production.

Therefore we tried to overcome this problem by increasing the amount of photosensitizer: the two systems tested (System B and C, Table 1) are characterized by an onium salt/photosensitizer ratio 3:1 and 1:1, *i.e.*, the photosensitizer concentration is around three and five times higher respectively. For both systems, the induction time is absent. The higher the amount of camphorquinone the higher the polymerization rate (Figure 2); in particular System C shows the same rate as System NH (which contains an amine as co-initiator) and reaches quantitative conversion in the same irradiation time.

These results put on evidence that excited CQ, if present in a sufficiently high concentration, is able to abstract hydrogen from other sources, first of all the monomer which contains labile abstractable hydrogens. This is an important result because the photoinitiator will be only a two component system and the detrimental use of an aromatic amine can be avoided.

### 2.2. Water content Effect

In the polymerization of restorative composites in oral cavities, water is always present. Therefore, the effect of the presence of water on the visible curing of our formulations was investigated.

Within a certain content, water acts towards cationic reactive propagating species as termination agent or chain transfer agent, enhancing the final epoxy group conversion. When the amount of water is too high, the nucleophilicity competitive effect with epoxy ring becomes important and a strong inhibition effect is induced: while the initial rate is not strongly affected, a very low final epoxy group conversion is achieved.

**Figure 3.**
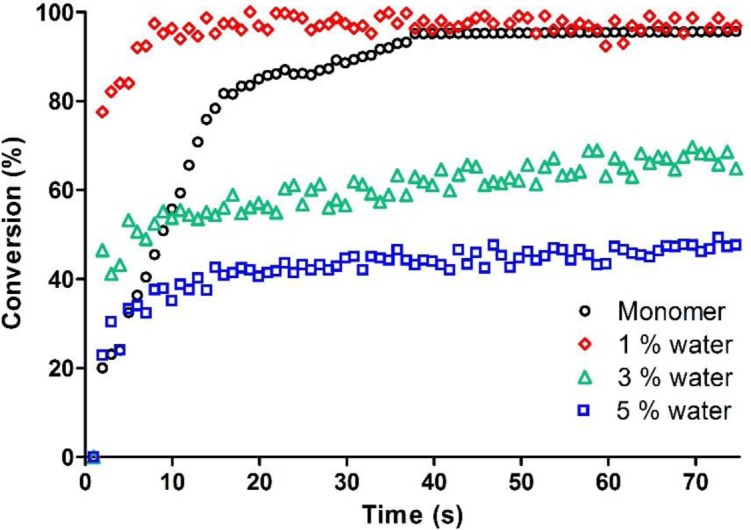
RT FT-IR conversion curves as a function of irradiation time for the epoxy based System C in presence of increasing amount of water.

In our study, water was added in the epoxy monomer in the range between 1 and 5 wt%. The kinetic curves of the System C previously described compared to the same formulation with an increasing amount of water are reported in Figure 3. They show that up to 1 wt% the water does not have any detrimental effect on photopolymerization rate and epoxy group conversion. This was also previously observed for vinyl ether systems [17]: it was shown that water begins to be detrimental for cationic curing process starting from 3 wt% in the photocurable formulation, with an important decrease on epoxy group conversion.

### 2.3. Filler Content Effect

Since the dental formulations contain a high inorganic filler content, the effect of SrO glass filler on the photopolymerization of the formulation without amine was studied.

The used filler was a Sr-(15%) and F-(2%)-containing very low refractive dental glass filler G018-163 (Schott) with a mean particle size of 1.5 μm, which was surface modified with 3-glycidoxypropyltrimethoxysilane. The glass fillers were silanized analogous to the method already reported in literature [18]. Typically, the glass fillers were silanized by mixing them with 1 wt% of water and 5 wt% of 3-glycidyloxypropyltrimethoxysilane at room temperature over a period of 2 h, and drying the modified fillers at 50 °C for 4 days.

**Figure 4.**
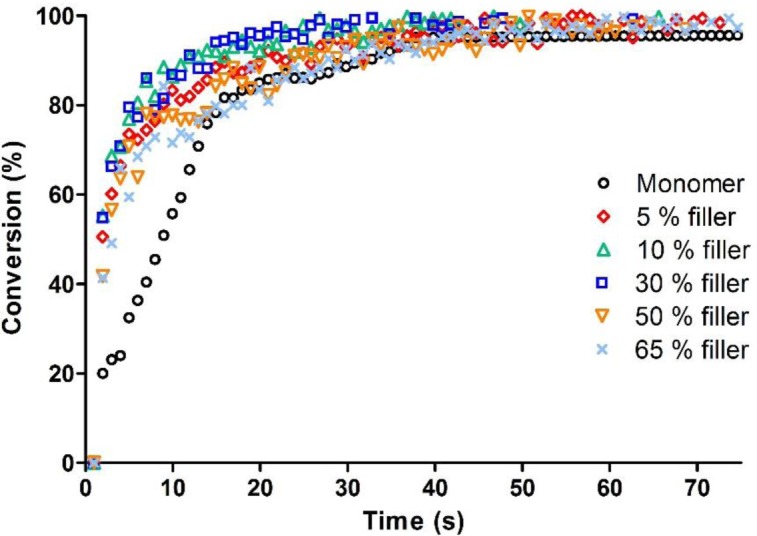
RT FT-IR conversion curves as a function of irradiation time for filled epoxy formulations.

The fillers were used in the range 5–65 wt%; the kinetic curves of the composites compared with the System C previously described are reported in Figure 4.

It is interesting to observe that the filler has negligible influence on the photopolymerization rate and nearly no effect on the epoxy group conversion, which is complete after 50 s of irradiation.

Photo-DSC experiments allowed to confirm the results obtained via real-time FT-IR, and show that the fillers do not have any influence on photopolymerization final conversion. The two high filler content formulations (containing respectively 50 and 65% of SrO glass filler) have similar exothermicity, indicating equal epoxy group conversion.

Furthermore, since the fillers are strongly interacting with the polymer network through their surface epoxy functional groups, we can expect materials with high mechanical performance.

## 3. Experimental Section

### 3.1. Materials

A silicone containing di-cycloaliphatic epoxy resin (bis[2-(3,4-epoxycyclohexyl)ethyl] tetramethyldisiloxane, UV30) was purchased from ABCR (Karlsruhe, Germany). Iodonium salt photoinitiator [4(1-methylethyl)phenyl][4-methylphenyl] iodonium tetrakis (pentafluorophenyl) borate, Rhodorsil 2074, was given by Rhodia (Lyon, France). Camphoquinone and ethyl 4-(dimethylamino)benzoate (EMBO) were purchased by Sigma-Aldrich (St. Louis, MO, USA). The chemical structures of the monomer and the photoinitiators are reported in Figure 1. Very low refractive dental glass filler, G018-163 (Schott), Sr-(15%) and F-(2%)-containing, with a mean particle size of 1.5 μm were used.

### 3.2. Curing Procedure and Characterization

In the case of pure cationic formulations, the defined photoinitiator system was directly added to the epoxy monomer. The filler was dispersed into the photocurable formulations using an Exakt three roll mill (Exakt Apparatebau, Norderstedt, Germany).

The kinetics of photopolymerization was determined by real-time Fourier Transform Infrared (FT-IR) spectroscopy, employing a Thermo-Nicolet 5700 (Thermo Scientific, Milano, Italy). The formulations were coated onto a silicon wafer forming 50 μm thick films. The samples were exposed simultaneously to blue light, which induces the polymerization, and to the IR beam, which analyzes *in situ* the extent of the reaction. Because the IR absorbance is proportional to the monomer concentration, conversion *versus* irradiation time profiles could be obtained. Epoxy group conversion was followed by monitoring the decrease in the absorbance due to epoxy groups in the region 760–780 cm^−1^.

An Astralis 5 (Ivoclar Vivadent, Naturno, BZ, Italy) gun lamp was employed to induce photocuring; the results are the average of up to 5 runs per each formulation investigated.

The Astralis 5 lamp was 75 W and 12 V and it was put at a distance of 2 cm from the sample.

A Mettler-Toledo DSC (Mettler-Toledo, Novate Milanese, MI, Italy) equipped with a robotic arm was used for thermal analysis. The equipment was calibrated using indium standards. Samples having masses of approximately 10 mg were cured in an open aluminum pans in nitrogen atmosphere. The DSC was used to study isothermal curing at 25 °C using the Astralis 5 gun-lamp for irradiation.

## 4. Conclusions

This paper reports for the first time the possibility to use composites based on cationic systems for dental restorative material applications. Important beneficial aspects such as lowering of shrinkage and therefore lowering of residual stress are expected.

An epoxy monomer cured by visible light was studied in this paper as a candidate material for dental applications. The cationic ring-opening polymerization activated via a radical-induced cationic process was investigated using a silicone containing dicycloaliphatic epoxy resin. We demonstrated that CQ is promoting the photopolymerization even in the absence of amines as hydrogen donor, thus showing to be able to abstract labile hydrogen from the epoxy monomer and give rise to the formation of carbon-centered radicals that are oxidized by the onium salt. A complete epoxy group conversion was reached after 50 s of irradiation with blue light.

By adding water to the photocurable formulation, up to a content of 1 wt%, it was not observed any detrimental effect on photopolymerization rate and epoxy group conversion.

In the presence of a high amount of filler (up to 65 wt% inorganic filler content), the epoxy resin showed a good reactivity. Both real-time FT-IR and photo-DSC analyses were in agreement, and a complete epoxy group conversion was achieved in all the investigated formulations.

## Figures and Tables

**Table 1.** Relative mixture compositions (wt%) for cationic systems.

Component	Formulation NH	Formulation A	Formulation B	Formulation C
UV30	0.96	0.96	0.96	0.96
Rhodorsil	0.031	0.036	0.030	0.020
CQ	0.003	0.004	0.010	0.020
EMBO	0.006	–	–	–
Rhodorsil : CQ (wt%)	10:1	9:1	3:1	1:1
